# Plasmacytoid Melanoma of the Urinary Bladder and Lymph Nodes with Immunohistochemical Expression of Plasma Cell Markers Revealing Primary Esophageal Melanoma

**DOI:** 10.1155/2012/916256

**Published:** 2012-10-24

**Authors:** Slim Charfi, Sameh Ellouze, Hela Mnif, Ali Amouri, Abdelmajid Khabir, Tahya Sellami-Boudawara

**Affiliations:** ^1^Department of Pathology, CHU Habib Bourguiba, CP 3029, Sfax, Tunisia; ^2^Department of Gastroenterology, CHU Hedi Chaker, CP 3029, Sfax, Tunisia

## Abstract

Plasmacytoid variant of melanoma is reported in only rare cases. We present the case of a 54-years-old man admitted for enlarged lymph nodes in the lumbar region. Initial diagnosis of plasmablastic lymphoma/plasma cell myeloma was considered. At our institute, a bladder polyp was removed. Microscopic exam demonstrated dense plasmacytoid cells infiltration with pigment deposits. Immunohistochemical study showed strong expression of HMB45, Melan A, and vimentin. There was focal positivity with S100 protein and CD138/syndecan-1. The diagnosis of metastatic plasmacytoid melanoma was finally established. Clinical exam revealed an esophageal melanoma with melanosis supporting its primary location. Although rarely, melanoma especially plasmacytoid variant may express plasma cell markers which may lead to erroneous diagnosis of plasma cell proliferation. Careful morphological examination for melanin pigment and the use of panel of melanocytic markers are helpful for diagnosis.

## 1. Introduction

Melanomas, particularly noncutaneous primaries and metastasis, are known to display tremendous pathological diversity which may mimic many other tumors [1]. This diversity includes cytomorphology, architecture, stromal component, and immunophenotype. Plasmacytoid variant of melanoma is reported in only rare cases [1–5]. Bladder metastasis of melanoma are extremely rare [6, 7]. To our knowledge, no bladder metastasis from a primary esophageal melanoma has been previously reported.

## 2. Case Presentation

**Figure 1:**
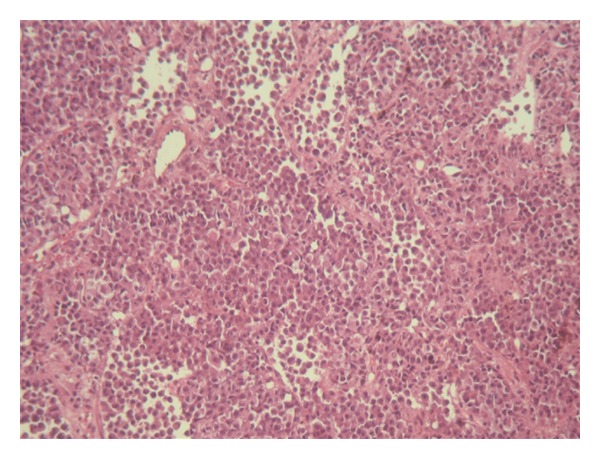
Diffuse proliferation of rounded neoplastic cells showing incohesion (HE ×40).

**Figure 2:**
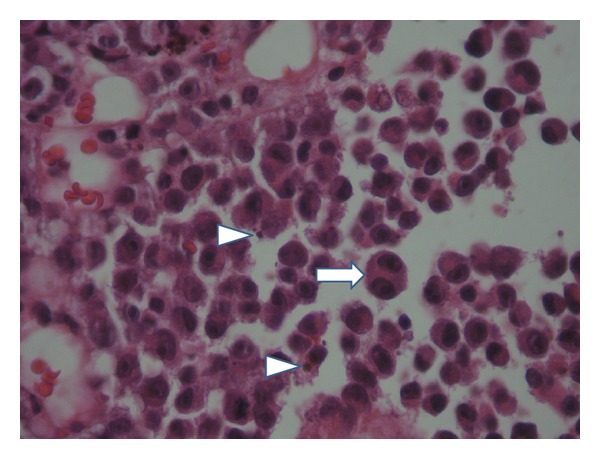
neoplastic cells demonstrated eosinophilic cytoplasm and eccentric nuclei with prominent nucleoli; some cells are binucleated (⇒); note the presence of pigment (⊷) (HE ×400).

**Figure 3:**
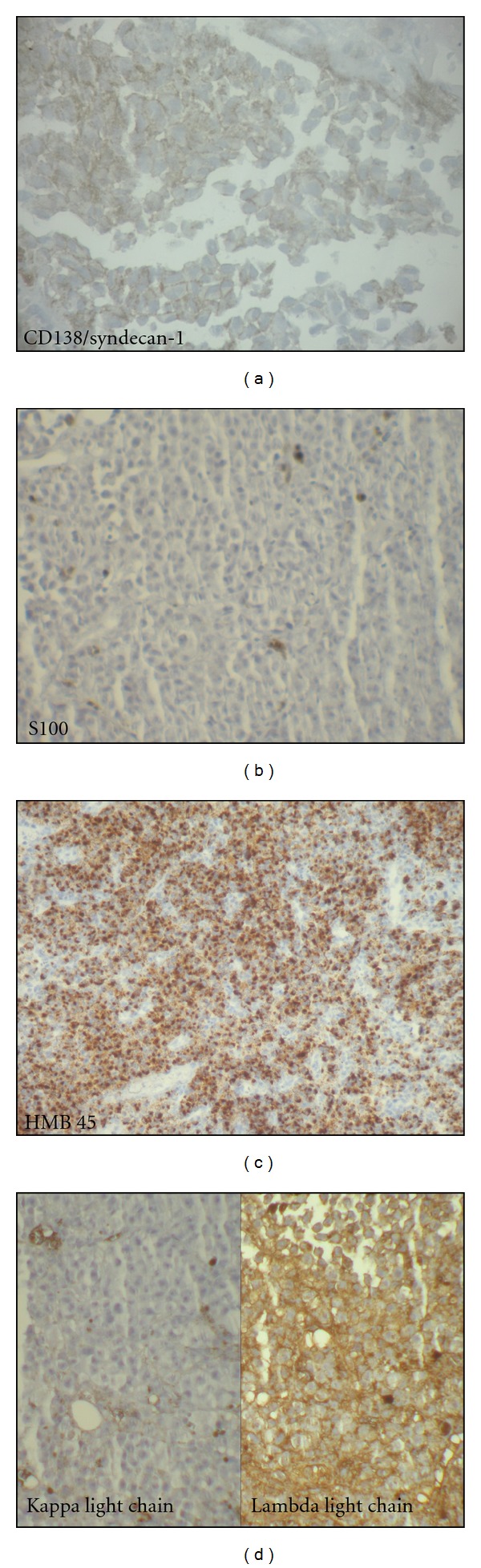
(a) Neoplastic cells exhibit immunoreactivity for CD138 (×100). (b) Very rare neoplastic cells are positive for S100 protein (×100). (c) Strong HMB45 expression by the neoplastic cells (×40). (d) Neoplastic cells are positive for lambda light chain and negative for kappa light chain (×100).

**Figure 4:**
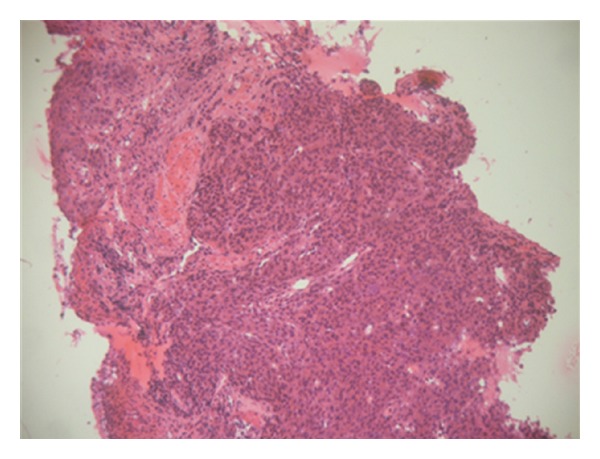
Esophageal tumor containing a mixture of epitheliod and spindle-shaped cells (HE ×40).

**Figure 5:**
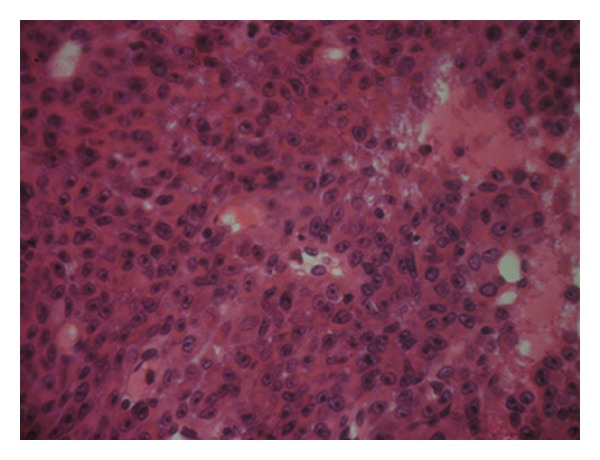
Esophageal tumor—sheets of large cells with plasmacytoid features (HE ×400).

**Figure 6:**
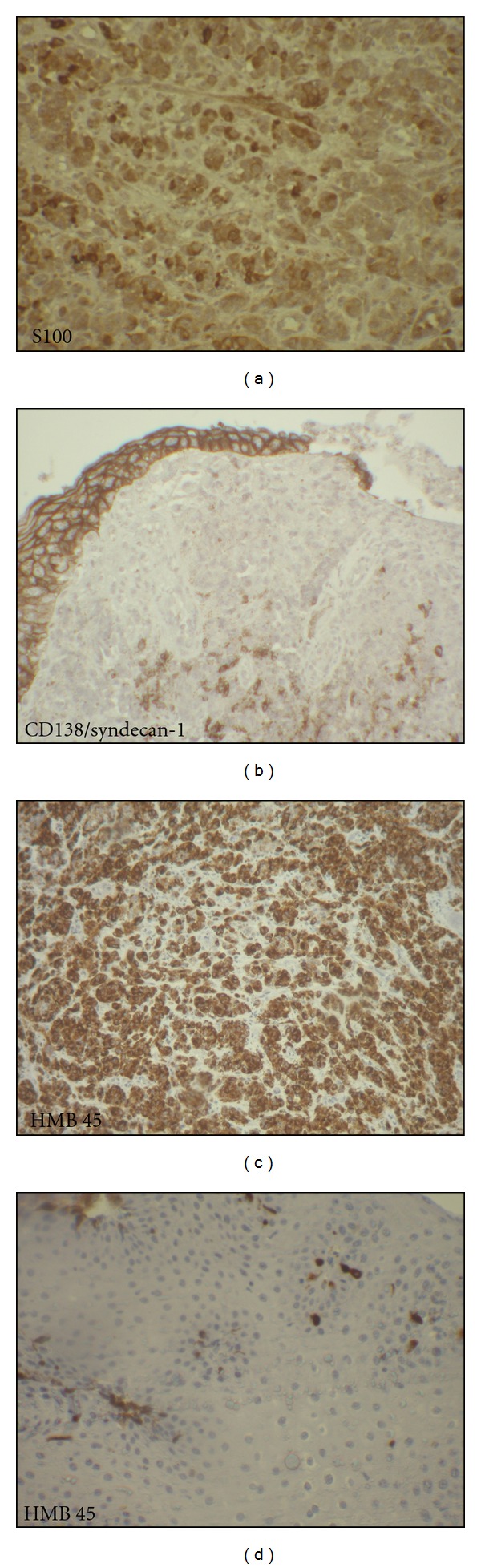
(a) Neoplastic cells showed strong positivity with S100 protein (×100). (b) Focal expression for CD138/syndecan-1 (×100). (c) Neoplastic cells showed strong positivity with HMB45 (×40). (d) HMB45 staining showing increased number of melanocytic cells at the basal layer of the squamous epithelium (×100).

The patient is a 54 years old man, with no medical history, admitted for investigation of enlarged lymph nodes of the lumbar region with a diagnosis of plasmablastic lymphoma/plasma cell myeloma. This diagnosis was established outside our institute on CT-scan lymph node biopsy. Initial pathologist described in his report a diffuse infiltration by plasmacytoid cells with immunohistochemistry expression of CD138/syndecan-1, MUM1, and immunoglobulin lambda light chain. Tumor cells were negative for S100 protein, kappa light chain, CD3, CD20, CD79a, and keratin KL1. HMB45 and Melan A were not tested. Laboratory analysis revealed an IgG lambda monoclonal immunoglobulin at immunofixation. The patient developed an acute renal failure. Cystoscopy exam demonstrated a 0,5 cm sessile bladder polyp which was removed. Microscopic exam showed a diffuse, dense, plasmacytoid cellular proliferation (Figure 1). Cells were small to medium with eosinophilic cytoplasm and eccentric nuclei with central prominent nucleoli. Some cells were pigmented (Figure 2). Tumor cells were strongly and diffusely positive for HMB45, Melan A, and vimentin. They were focally positive for S100 Protein, CD138/syndecan-1, and immunoglobulin lambda light chain (Figure 3). Tumor cells were negative for keratin AE1/AE3, keratin 7, keratin 20, epithelial membrane antigen (EMA), CD79a, and immunoglobulin kappa light chain (Table 1). MUM1 was not available at our department. Thus, the diagnosis was redressed to metastatic plasmacytoid melanoma. Microscopic examination of bone morrow was unremarkable. The patient underwent an upper endoscopy, which revealed a 2 cm, lobulated, and pigmented mass located in the junction medium-distal esophagus. Biopsy of this mass demonstrated a tumor proliferation containing a mixture of epithelioid and spindle-shaped cells arranged in fascicles with presence of melanin pigment (Figure 4). There were some cells with plasmacytoid feature (Figure 5). Immunohistochemically, tumor cells were positive for HMB45, Melan A, S100 protein, and only focally for CD138/syndecan-1. HMB45 staining showed an increased number of melanocytes at the basal layer of the squamous epithelium (Figure 6) suggesting the presence of melanosis and furthermore that this location represents the primary melanoma. The patient died one month after the final diagnosis.

## 3. Discussion

Plasmacytoid variant of melanoma is a rare finding [1–5, 8] which may mimic many other entities especially plasma cell proliferation. The use of immunohistochemistry in the diagnosis of such tumors is primordial. However, as shown in our case, this study may lead to erroneous diagnosis. In fact, we observed a positive staining in primary oesophageal and metastasis melanoma with the plasma cell markers CD138/syndecan-1, MUM1, and immunoglobulin lambda light chain. The expression of CD138/syndecan-1 in melanoma is reported in only one case [8]. It consists of a large ulcerated cutaneous melanoma which was initially considered as extramedullary plasmocytoma. CD138/syndecan-1 is a heparin sulphate bearing proteoglycan. CD138/syndecan-1 plays an important role in plasma cell differentiation and functions in adhesion and motility of cells. It is expressed on most of the myeloma tumour cells and cells of certain other tumors of b lineage and also on epithelial cells [9]. MUM1 is a member of the interferon regulatory factor family of transcription factors. In hematolymphoid system, MUM1 plays a significant role in terminal B-cell differentiation and hence is a potentially specific marker for plasmacytic differentiation [10]. MUM1 is present in a wide spectrum of hematolymphoid neoplasms and in malignant melanomas but is absent in the other human neoplasms [10, 11]. The study of Sundram et al. has shown that MUM1 is more sensitive than both HMB45 and Melan A in cases of conventional primary and metastatic melanomas. MUM1 was also positive for some cases that were weakly positive with S100 protein [10]. Shanks and Banerjee reported the expression of another plasma cell marker VS38 in melanoma [12]. The immunohistochemical expression of lambda light chain in our case was associated to the detection of IgG lambda monoclonal immunoglobulin in serum immunofixation. Immunoglobulin light chain restriction by flow cytometry of tumor cells was also reported by Lehmer et al. [8]. The signification of the expression of plasma cell markers and their role in the biologic behaviour of melanocytic tumors are unclear and may need additional studies for clarification. We think that these findings represent a probably aberration in immunophenotypic melanoma. Lehmer el al. suggested that neoplastic plasma cells are associated to melanoma participating in its chronic inflammatory [8]. Immunohistochemical aberrant findings in melanoma are well known. The most frequent of these is cytokeratin which may expressed up to 10% in melanoma. Expression of neuroendocrine markers has also been reported [1, 5].

Our case showed an immunophenotypic heterogeneity of S100 protein expression between primary esophageal and metastasis locations. S100 protein is expressed in 94% and 95% of primary and metastatic melanomas, respectively [13]. Only 3-4% of melanomas were S100 protein negative. Morphologically, these melanomas present as in our case are atypical features (signet cell, rhabdoid, etc.) [1, 5].

Bladder metastasis from melanoma is a very rare finding. Less than 10 cases were reported in the English literature. Cutaneous melanoma represents most primary location [6, 7]. To our knowledge, bladder metastasis from primary esophageal melanoma was not previously reported.

Primary esophageal melanoma represents 0.1 to 0.5% of the primary malignant esophageal neoplasms and approximately 0.5% of melanoma originate in the esophagus. Cutaneous malignant melanomas metastatic to the esophageal are more common than primary esophageal melanoma [14]. The histopathologic criteria for diagnosing primary esophageal melanoma have not been clearly established. Sanchez et al. reported that in situ melanoma is the most consistent criterion. However, this criterion is rarely present, as in our cases, in mucosal biopsies [15]. Two additional criteria have been proposed by Sabanathan et al.: presence of esophageal melanocytosis and a diagnosis of exclusion. Melanocytosis is characterized by the presence of increased melanocytes in the basal layer of esophageal squamous mucosa, and the melanocytes do not show atypia [14].

In Conclusion, this paper emphasises that plasma cell markers are not entirely specific and are particularly expressed in melanoma. We recommend a panel of immunohistochemical markers which should include more than one melanocytic marker to exclude malignant melanoma, even if the tumour has a plasmacytoid appearance suggestive of plasma cell proliferation. In addition, careful morphological assessment and particularly the research of melanin pigment reduce the risk of erroneous interpretation of aberrant results.

## Figures and Tables

**Table 1:** Panel of immunohistochemical stains.

Antibody	Clone	Company	Dilution
Keratin	AE1/AE3	DAKO	1 : 50
CD138	MI15	DAKO	1 : 50
MelanA	A103	DAKO	1 : 50
Vimentin	V9	DAKO	1 : 50
HMB45	HMB45	DAKO	1 : 50
Lambda immunoglobulin light chain	A0193	DAKO	1 : 2000
Kappa immunoglobulin light chain	A0191	DAKO	1 : 2000
Keratin 7	OV-TL 12/30	DAKO	1 : 50
Keratin 20	Ks20.8	DAKO	1 : 50
EMA	E29	DAKO	1 : 100
CD79a	JCB117	DAKO	1 : 50
